# Continued cannabis use at one year follow up is associated with elevated mood and lower global functioning in bipolar I disorder

**DOI:** 10.1186/s12888-015-0389-x

**Published:** 2015-02-05

**Authors:** Levi Roestad Kvitland, Ingrid Melle, Sofie Ragnhild Aminoff, Christine Demmo, Trine Vik Lagerberg, Ole Andreas Andreassen, Petter Andreas Ringen

**Affiliations:** 1 NORMENT, KG Jebsen Center for Psychosis Research, Division of Mental Health and Addiction, Oslo University Hospital and Institute of Clinical Medicine, University of Oslo, Norway TOP Study, Building 49, Oslo University Hospital, Ullevål, Kirkeveien 166, PO Box 4956 Nydalen, 0424, Oslo, Norway; 2 Division of Mental Health Services, Department of Specialized Inpatient Treatment, Akershus University Hospital, Akershus, Norway; 3 Division of Mental Health and Addiction, Oslo University Hospital, Oslo, Norway

**Keywords:** Cannabis, Bipolar

## Abstract

**Background:**

There is limited knowledge about how environmental factors affect the course of bipolar disorder (BD). Cannabis has been proposed as a potential risk factor for poorer course of illness, but the role of cannabis use has not been studied in a first treatment BD I sample.

**Methods:**

The present study examines the associations between course of illness in first treatment BD I and continued cannabis use, from baseline to one year follow up. Patients (N = 62) with first treatment DSM-IV BD I were included as part of the Thematically Organized Psychosis study (TOP), and completed interviews and self-report questionnaires at both baseline and follow up. Cannabis use within the last six months at baseline and use between baseline and follow up (“continued use”) was recorded.

**Results:**

After controlling for confounders, continued cannabis use was significantly associated with elevated mood (YMRS) and inferior global functioning (GAF-F) at follow up. Elevated mood mediated the effect of cannabis use on global functioning.

**Conclusions:**

These results suggest that cannabis use has clinical implications for the early course of BD by increasing mood level. More focus on reducing cannabis use in clinical settings seems to be useful for improving outcome in early phase of the disorder.

## Background

The prevalence of substance use in bipolar disorder (BD) is high [1-3] with cannabis being the most commonly used drug [3]. This is of interest since cannabis has been indicated as a risk factor for developing BD [4-6]. Cannabis use has also been associated with severity indicators in chronic BD, including earlier age of onset for the first affective episode [7-13], increased risk of manic episodes [4,14], prolonged duration of episodes [4-6], switch to mania in depressed individuals [15] and increased suicidal ideation and suicide risk [16,17], in addition to a more severe general course of the illness [18-20]. Cannabis abuse has furthermore been shown to predict poorer medication adherence in BD patients [21]. Most of these studies have included patients mainly in the chronic phase of illness after multiple mood episodes, and we cannot rule out the possibility that these findings are biased by a selection of patients with a more severe course, possibly being more prone to self-medication with cannabis.

Longitudinal studies of BD samples recruited at first treatment are very few. Two follow-up studies of first time hospitalized patients with BD I indicate that patients with cannabis use spend more time in affective episodes and exhibit more rapid cycling over the first year of treatment [22], and that periods with cannabis use coincide with periods with manic and hypomanic episodes over a mean follow-up period of 4.5 years [6]. Both studies suggest that continued cannabis use in patients with recently diagnosed BD has a negative impact on the course of illness. Since these two studies are based on hospitalized patients in two university hospitals with well-acknowledged BD research groups, their patient samples might be biased towards patients with a higher severity of illness.

The current study is based on patients recruited during their first adequate treatment for a manic episode from both inpatient and outpatient services in a catchment area based treatment system, and subsequently followed up after one year. We here aim to ascertain the rate of continued cannabis use over the first year of treatment in patients with recent onset BD and identify clinical outcomes associated with continued use, by exploring the relationship between cannabis use patterns over the follow-up period and clinical status at one year follow-up.

## Methods

One hundred and one patients with recent onset DSM-IV [23] BD-I were recruited consecutively from 2003 until 2013 from in- and outpatient units at all major hospitals in the Oslo area as a part of The Thematically Organized Psychosis (TOP) Study at the University of Oslo and Oslo University Hospital. The patients had both psychotic and non-psychotic forms of bipolar disorder. Out of these, 62 patients (63%) participated in a personal follow-up examination after one year. Of the 39 patients that did not attend the follow-up, 20 had decided to withdraw from the study, 18 had moved and were impossible to reach and one patient had died. There were no significant differences in baseline demographic and clinical characteristics between follow-up participants and study drop-outs.

The full inclusion criteria were as follows: meeting DSM-IV diagnostic criteria for BD I, being within the first year of receiving adequate treatment for a manic episode, age between 17 – 65 years. Patients were excluded from the study if they had pronounced cognitive deficits (IQ lower than 70), a neurological disorder, moderate/severe head injury, or were not able to speak a Scandinavian language or to give written informed consent. The patients were given both oral and written descriptions of the study before consenting to participate. The study was approved by the Regional Committee for Medical Research Ethics and the Norwegian Data Inspectorate.

### Assessments

Patients referred to the study were interviewed by trained research fellows (psychologists and medical doctors). Diagnosis and episodes of illness were determined at baseline using the Structural Clinical Interview for DSM-IV Axis I disorders (SCID module I, chapters A-E) [24], with the aid of medical charts. For more details, see [1]. Patients were interviewed in detail, based on a common semi-structural interview form, about substance use in the six months prior to inclusion and in the follow-up period. Forty-seven patients did not use cannabis at any point during this period, 7 patients used cannabis at baseline but not at follow-up, 2 patients started to use cannabis in the follow-up period while 6 patients used cannabis at both time points. Based on these data, the sample was divided into those with continued cannabis use (defined as any cannabis use at both time points, n = 6) and those without continued use (the rest of the patient group, i.e. both non-users and those using at one but not both time-points, n = 56). The continued use patients reported an average use of cannabis at both baseline and follow up of 2–3 times a week.

Global functioning was measured using the functioning part of the Global Assessment Functioning scale split version (GAF-F) [25,26]. Cut-off for functional recovery was set at a GAF score of 61 [27]. Current depressive symptoms were assessed with the Inventory of Depressive Symptoms – Clinician rated (IDS-C) [28], current manic symptoms were rated with the Young Mania Rating Scale [29], and current psychotic symptoms were assessed with the positive symptoms subscale of the Positive And Negative Syndrome Scale (PANSS) [30] at both time points.

Medication and socio-demographic data were obtained by clinical interviews supported by medical chart information. Premorbid functioning was measured with the Premorbid Adjustment Scale (PAS), divided into academic and social functioning [31]. Childhood premorbid functioning was chosen due to the young age of the sample. Symptomatic recovery was defined as a lack of affective and psychotic symptoms the previous 6 months (PANSS-P less than 4 and no mood episodes as verified by the SCID).

All clinical personnel completed a training program in diagnostics (SCID) and symptom rating (PANSS), based on the training program at the University of California, Los Angeles. They also attended bi-weekly diagnostic consensus meetings led by experienced clinicians in the field of severe mental illness diagnostics. The inter-rater reliability was good with an overall kappa-score of 0.77 (95% LI [0.60, 0.94]) for diagnoses and ICCs of 0.82 [32] for PANSS positive symptoms and 0.86 for the GAF [1].

### Statistical analyses

The statistical package for the social sciences (SPSS) version 20.0 (SPSS Inc, Chicago, IL, USA) was used for statistical analyses. Group comparisons for continuous variables were evaluated with independent sample T-tests, and group comparisons for dichotomous data were evaluated with Chi-squared tests or Fischer’s exact tests as appropriate. Level of significance was set to p < 0.05, two-sided. The overall effects of continued cannabis use on key baseline measures and on outcome measures at one-year follow-up were first evaluated with a multivariate analysis of variance (MANOVA) with continued cannabis use as the fixed factor. Outcome dimensions indicated to be different between continued cannabis users and the rest of the patients through the MANOVA, were followed up with a series of hierarchical block-wise multiple linear regressions analyses controlling for possible confounders of this association. In addition to sex and age, variables with strong correlations with both continued use of cannabis and the outcome measure were selected as possible confounders, based on a bivariate analysis. Premorbid academic functioning (PAS) was added to the models in order to investigate the role of premorbid traits in the associations. Hence the baseline measures of YMRS and GAF-F were entered in the first step, age and gender in the second, premorbid functioning in the third step and continued cannabis use in the fourth step of the model. There were no associations between the outcome measures and drug treatment adherence on continued cocaine, amphetamine or alcohol use. These factors were thus not added to the model. Information about patient hospitalisations were not collected, and thus not corrected for. Since global functioning (GAF-F) is highly correlated with mood symptoms, a separate analysis was performed with YMRS at one-year follow up in the second-to-last step. To evaluate the possibility of outliers mediating the main effect, a scatterplot of GAF-F by YMRS scores was performed and examined. Finally, in order to investigate if current cannabis use influenced the results, follow-up analyses were done removing patients with cannabis use at one time-point, but not the other, from the no continued cannabis use group, thus contrasting the continuous cannabis users from the non-users.

## Results

The mean age of the sample was 30.9 years (SD: 9.9 years), and 37 patients (60%) were female. Lifetime cannabis use was reported in 52% of the sample. There were more males in the continued use group (p < .05) than in the group without continued use. There were no significant differences in key clinical characteristics at baseline, including YMRS and GAF-F levels. There was a negative association between YMRS and GAF-F, indicating that patients with high levels of elevated mood had poorer functioning. The groups did not differ in any other features (Table 1), including the number in symptomatic remission at follow-up (3 (50%) in the continued cannabis use group vs 38 (68%) in the no continued cannabis use group, p = .601). Four (67%) of the patients in the continued cannabis use group had not reached a level of functional recovery compared to 19 (29%) patients in the group without continued use; however, this difference was not statistically significant (p = .390).

**Table 1:** **Demographic and clinical characteristics of patients with- and without continued cannabis use at one year follow-up**

	No continued cannabis use			Continued cannabis use			
	N = 56			N = 6			
	N	Mean	SD	N	Mean	SD	p
Age (years)	56	32.3	10.0	6	30.5	9.7	**.**691
IDS total score	53*	11.2	10.0	6	18.5	11.9	.198
YMRS total score	56	2.3	4.0	6	7.3	5.5	.076
PANSS positive total score	56	8.8	2.6	6	9.7	2.6	.439
GAF-F	56	65.3	16.0	6	49.0	16.1	.056
	N	%		N	%		P
Females	36	64.3		1	16.7		.**035**
Current use of antipsychotic or mood stabilizing medication	42	79.2		4	66.7		.605

*Missing data.

IDS = Inventory of depression Scale; YMRS = Young Mania Rating Scale; PANSS-P = Positive and Negative Syndrome Scale-Psychotic; GAF-F = Global assessment of functioning.

The MANOVA indicated that the continued use group experienced significantly elevated mood, as measured by the YMRS, and significantly lower global functioning as measured by the GAF-F compared to the group without continued use. The effect size for the difference in GAF-F was high. There were however no significant differences in the levels of depression or psychotic symptoms between the groups (Table 2). Repeating the analyses, this time only contrasting continued cannabis users with non-users, gave the same findings.

**Table 2:** **Levels of symptoms and functioning in patients with and without continued cannabis use at one year follow-up (MANOVA between-subject effects)**

	No continued cannabis use N = 56		Continued cannabis use N = 6					
Measure	Mean	SD	Mean	SD	Mean square	F	P	*d*
GAF-F	66.02	15.31	49.00	16.11	1561.12	6.599	**.013**	1.4
PANSS-P	8.70	2.55	9.67	2.58	5.05	.774	.383	0.9
IDS	11.17	9.89	18.50	11.89	289.60	2.802	.100	0.1
YMRS	2.25	3.90	7.33	5.47	139.53	8.469	**.005**	−0.3
*Wilks’ λ 3.299 p = .017*								

GAF-F = Global assessment of functioning; PANSS-P = Positive and Negative Syndrome Scale-Psychotic; IDS = Inventory of depression Scale; YMRS = Young Mania Rating Scale. Significant values in boldface.

**Figure 1:**
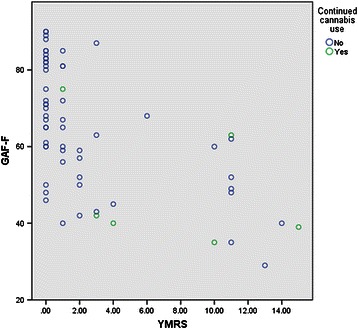
**GAF-F by YMRS with or without continued cannabis use.**

The bivariate analysis showed a correlation between continued cannabis use and sex (−.287 p < .05), and a negative correlation between YMRS at baseline and GAF-F at baseline (−.347 p < .01), and between sex and premorbid academic functioning (.357 p < .01). In the regression analysis of elevated mood (Table 3), the initial level of elevated mood contributed significantly and with an even stronger association between elevated mood at follow-up and continued cannabis use than indicated by the bivariate analysis. After controlling for possible confounders of the relationship between global functioning at follow-up and continued cannabis use (Table 4), we found that baseline global functioning and continued cannabis use both contributed significantly in the final model (4A). When correcting for level of elevated mood (YMRS scores) at the second to last step, the impact of continued cannabis use was reduced to a trend level of significance (4B). Gender, age and premorbid functioning (as represented by the childhood level of academic functioning in the presented final model) did not contribute significantly to any of the models. The scatterplot did not indicate any outliers (Figure 1).

**Table 3:** **Multiple regression analysis with elevated mood (YMRS scores) at 1 year as dependent variable**

	Block model summary for each step		Contribution of separate variables for last step				
Block no. variable	R2 change	F change	Beta	t	P Value	95% CI of B	
						Lower	Upper
Constant	…	…		2.630	.011	.423	3.111
1	.098	6.526					
YMRS (Baseline)			.313	2.555	.**013**	.047	.389
2	.024	.791					
Sex			-.076	-.604	.548	−2.886	1.547
Age			-.152	−1.201	.235	-.179	.045
3	.042	2.862					
Premorbid childhood academic functioning			.212	−1.629	**.**096	-.140	1.664
4	.115	8.913					
Continued use of cannabis			.360	2.985	**.004**	1.729	8.781

Total model: F 4.329 p = .002. Adj r^2^ .214.

**Table 4:** **Multiple regression analysis with GAF-F at one year follow-up as dependent variable**

	Block model summary for each step		Contribution of separate variables for last step				
Block no. variable	R2 change	F change	Beta	t	P Value	95% CI of B	
						Lower	Upper
Constant	…	…		4.432	.000	22.543	59.633
1	.095	6.270					
GAF-F (Baseline)			.308	2.504	.**015**	.087	.779
2	.024	.780					
Sex			-.158	−1.247	.217	−13.794	3.202
Age			-.020	-.162	.872	-.461	.392
3	.028	1.895					
Premorbid childhood academic functioning			-.178	−1.377	.174	5.997	1.110
Model A							
4	.119	9.052					
Continued use of cannabis			-.363	−3.009	**.004**	−33.745	−6.770
Model B							
4	.277	26.934					
YMRS (at 1 yr)			-.565	-.5190	**.000**	−2.991	−1.325
5	.032	3.224					
Continued use of cannabis			-.200	−1.796	.078	−23.582	1.293

Total model: A F 4.047 p = .003. Adj r^2^ .200.

Total model: B F 7.675 p = .000. Adj r^2^ .456.

## Discussion

The main result of the current study is that continued cannabis use had a statistically significant association with elevated mood but not with depressive or psychotic symptoms, over a one year follow-up period in a sample of first treatment BD-I patients. This relationship was not explained by differences in age, gender, premorbid functioning or symptoms at baseline. Continued cannabis use also had a significant association with poorer global functioning, but this association seemed to be mediated by the elevated mood levels.

The finding of an association between continued cannabis use and elevated mood confirms findings from the two existing first-hospitalization samples in addition to several multi-episode samples [2,6,14,19,20,22,33,34] and supports the hypothesis that cannabis use in these groups is particularly associated with a higher risk for elevated mood [3-6]. The association was not fully explained by baseline levels of mood symptoms. Furthermore, premorbid functioning did not contribute to the explanation, contrary to findings in schizophrenia [35,36].

The finding of worse global functioning in the continued cannabis group is in line with previous studies of multi-episode inpatients with BD [20,33] and negative effects of continued cannabis use in general in BD [37,38]. Our findings indicate that the reduction in global function is partly mediated by elevated mood. The lack of an association between continued cannabis use and depression is in line with a recent systematic review and meta-analysis of mainly multi-episode samples [39]. The lack of an association with psychotic features is slightly surprising given the extensive amount of studies on the relationship between cannabis and psychosis risk [40-43], but we cannot rule out a type II error due the low number of continued cannabis users, and this finding warrants further research. The YMRS contains items of psychotic manic symptoms [29]. The lack of an association between continued cannabis use and psychotic features could strengthen the notion of a primary association to mood symptoms since this indicates that the higher YMRS scores are not mainly based on psychosis-related items [29], a view supported by the findings of an association between poorer global functioning and elevated mood also at baseline. The effect of continued cannabis use on outcome measures after 12 months did not seem to be explained by premorbid traits, further strengthening a hypothesis of a direct association between cannabis use and outcome.

### Strengths and limitations

The main strength of the study is the well characterized and relatively large prospective sample of patients followed during one year after the first treatment for mania in BD-I. The catchment area based consecutive sampling procedure including both in-and outpatient treatments services gives the sample a high degree of representativity.

The low number of continued use patients is our main limitation, but is unlikely to induce type I errors since results did not appear to be driven by outliers. However, type II errors cannot be ruled out. This is a longitudinal study with two cross-sectional points of assessment; we thus lack reliable data of temporal sequencing of cannabis use and mood symptoms in the follow up period. It is thus not possible to conclude with certainty which phenomenon that drives the other. Information about cannabis use was based on self-reports. Self-reports of substance use have however been shown to have considerable validity in earlier studies [44-46].

## Conclusion

In conclusion, the current study showed that patients with continued cannabis use throughout the first year of treatment of BD I were at higher risk for elevated mood and worse global functioning compared to the patients without continued cannabis use. The poorer functioning seemed to be, at least in part, mediated by the elevated mood. These findings indicate that continued cannabis use, also below the threshold for a DSM-IV diagnosis of abuse or dependency, may have important clinical implications for patients suffering from BD-I. Future research should aim at replicate these findings in a larger sample to minimize the risk of type II errors.
